# Thermotolerant coral–algal mutualisms maintain high rates of nutrient transfer while exposed to heat stress

**DOI:** 10.1098/rspb.2023.1403

**Published:** 2023-09-20

**Authors:** Dustin W. Kemp, Kenneth D. Hoadley, Allison M. Lewis, Drew C. Wham, Robin T. Smith, Mark E. Warner, Todd C. LaJeunesse

**Affiliations:** ^1^ Department of Biology, University of Alabama at Birmingham, AL, USA; ^2^ Department of Biological Sciences, University of Alabama, AL, USA; ^3^ Department of Biology, Pennsylvania State University, University Park, PA, USA; ^4^ Center for Marine and Environmental Studies, University of the Virgin Islands, St. Thomas, VI, USA; ^5^ School of Marine Science and Policy, University of Delaware, Lewes, DE, USA

**Keywords:** coral reefs, physiological trade-offs, global warming, mutualistic symbiosis, nutrient cycling

## Abstract

Symbiotic mutualisms are essential to ecosystems and numerous species across the tree of life. For reef-building corals, the benefits of their association with endosymbiotic dinoflagellates differ within and across taxa, and nutrient exchange between these partners is influenced by environmental conditions. Furthermore, it is widely assumed that corals associated with symbionts in the genus *Durusdinium* tolerate high thermal stress at the expense of lower nutrient exchange to support coral growth. We traced both inorganic carbon (H^13^CO_3_^–^) and nitrate (^15^NO_3_^–^) uptake by divergent symbiont species and quantified nutrient transfer to the host coral under normal temperatures as well as in colonies exposed to high thermal stress. Colonies representative of diverse coral taxa associated with *Durusdinium trenchii* or *Cladocopium* spp. exhibited similar nutrient exchange under ambient conditions. By contrast, heat-exposed colonies with *D. trenchii* experienced less physiological stress than conspecifics with *Cladocopium* spp. while high carbon assimilation and nutrient transfer to the host was maintained. This discovery differs from the prevailing notion that these mutualisms inevitably suffer trade-offs in physiological performance. These findings emphasize that many host–symbiont combinations adapted to high-temperature equatorial environments are high-functioning mutualisms; and why their increased prevalence is likely to be important to the future productivity and stability of coral reef ecosystems.

## 1. Introduction

The process of reef-building and the creation of coral reef ecosystems relies on mutualistic symbioses between calcifying cnidarians and dinoflagellates (family: Symbiodiniaceae). Through photosynthesis and absorption of waste nitrogen from the host, symbiotic dinoflagellates transform inorganic carbon and nitrogen to organic molecules (e.g. carbohydrates, lipids and amino acids) and transfer these products to support and promote the growth and health of the coral colony. This exchange of nutrients is influenced by intrinsic and extrinsic factors, which are important to the overall performance of the mutualism. While external conditions such as light intensity, temperature, *p*CO_2_ and eutrophication can influence symbiont physiology and alter the relative benefits to the host [1–3], the identity of the symbiont can have a large effect on how a particular partnership functions under various circumstances. Therefore, partnerships better adapted to prevailing environmental conditions are fundamental for the productivity and persistence of corals and the ecosystem they construct over geological time scales [4].

Host–symbiont combinations vary at local and regional spatial scales [5–8]. The number of different host–symbiont pairings is influenced by partner specificity, how symbionts are acquired from generation to generation (i.e. horizontally or vertically transferred), and by major environmental factors such as irradiance and temperature [9–12]. This variation in host–symbiont pairings enhances reef coral resilience to anthropogenic climate change. Thermal stress from recurring marine heat waves causes many coral–dinoflagellate mutualisms to destabilize, initiating episodes of mass coral bleaching where colonies lose most of their symbionts; and when severe or prolonged, it leads to mass mortality [13,14]. Despite this sensitivity, there are certain coral–dinoflagellate combinations that endure episodic marine heatwaves [5,15–17].

Coral populations thriving in unusually warm near-shore or lagoonal habitats, tend to harbour symbiont species different from what is found in coral communities living in nearby offshore reefs with cooler open ocean waters [5,8,17,18]. Symbionts in the genus *Durusdinium* are notably adapted to warm or widely fluctuating temperatures [19]. Associations with *Durusdinium* spp. are often resistant to thermal stress and colonies hosting these symbionts tend to maintain stability at temperatures that are generally 1–2°C higher compared with colonies with other symbiont species [17,20–22]. However, this thermal tolerance may come at a steep metabolic cost to the animal, leading to a reduction in its growth and reproductive capabilities [23–26].

Experiments have shown that *Acropora millepora* and *A. tenuis* from the Great Barrier Reef experience reduced growth and nutrient translocation when associated with *Durusdinium* instead of *Cladocopium* [23,24,27]. These findings support the notion that symbioses with *Durusdinium* may result in physiological trade-offs for coral colonies with this symbiont. However, ecological context of coral–Symbiodiniaceae associations is shaped by long-term evolutionary processes that can influence their physiology. In equatorial regions of the Indo-west Pacific, corals have been co-evolving with *Durusdinium* dinoflagellates since the Pleistocene and these mutualisms are widespread [5,19]. Working on coral communities in Palau, isotopic labelling was used to quantify inorganic carbon and nitrogen assimilation by the symbiont and subsequent transfer to coral host tissue and skeleton in colonies of diverse reef-building corals associated with either *Durusdinium trenchii* or *Cladocopium* spp. Experimental heating was then applied to measure the influence of thermal stress on nutrient transfer. This research aims to investigate the existence of physiological trade-offs in hosts associated with *Durusdinium trenchii* in regions where mutualisms with this symbiont are abundant. These findings provide further support for the importance of continued nutrient exchange in the maintenance of coral–dinoflagellate mutualisms exposed to thermal stress.

## 2. Material and methods

### (a) Coral collection

Corals from Rebotel Reef on the western barrier reef of Palau (7.2497°N, 134.2288°E) were collected for offshore samples, while near-shore corals were collected in Nikko Bay (7.3243°N, 134.4936°E) approximately 28 km away. The corals *Acropora muricata* and *Coelastrea aspera* were sampled in March of 2014 from both locations and used in the initial thermal experiments. Two additional coral species, *Pachyseris rugosa* and *Cyphastrea chalcidicum*, were sampled from the same locations and treated the same way in March of 2015. A total of eight colonies of each species were collected using a hammer and chisel at a depth of 5–10 m (offshore) or 1–5 m (near-shore) to ensure similar irradiance conditions, and each colony was sampled a minimum distance of 10 m from surrounding colonies to better ensure sampling unique coral genets. While thermal experiments were conducted in 2014 and 2015 the thermal histories and light levels indicate similar conditions during this time period and allowed physiological comparisons across host species and population origin [28]. Colonies were transported to the Palau International Coral Research Center (PICRC) and fragmented into replicate pieces (clone ramets) and placed into a 1200 l flow-through aquariums supplied with natural seawater and held at 27.5°C. Corals were allowed to heal for a minimum of 2 days and were then placed on individual 5 cm^2^ PVC tiles with marine epoxy (splash zone compound A-788) and returned to the holding aquariums for 12–16 days to recover before the start of the experiment.

### (b) Experimental system

During the experiment, each treatment system consisted of 12 plastic treatment bins, each with a capacity of 56 l, connected to a central sump with a capacity of around 1200 l. The seawater in the sump was either heated or maintained at a control temperature before being sent to the treatment bins. The control temperature of 27.5°C was maintained using a chiller system, while high-temperature treatments of 32°C were achieved using titanium heating elements. All sumps and experimental tanks were supplied by seawater collected directly off a nearby pier at a depth of 3 m and then passed through a pressurized sand filter and aquarium filter pads to minimize particulate material. Water was distributed to each experimental tank with the flow-through rate of approximately 120 l h^−1^ resulting in complete seawater turn-over time every 15–20 min and minimized any evaporation.

**Figure 1.**
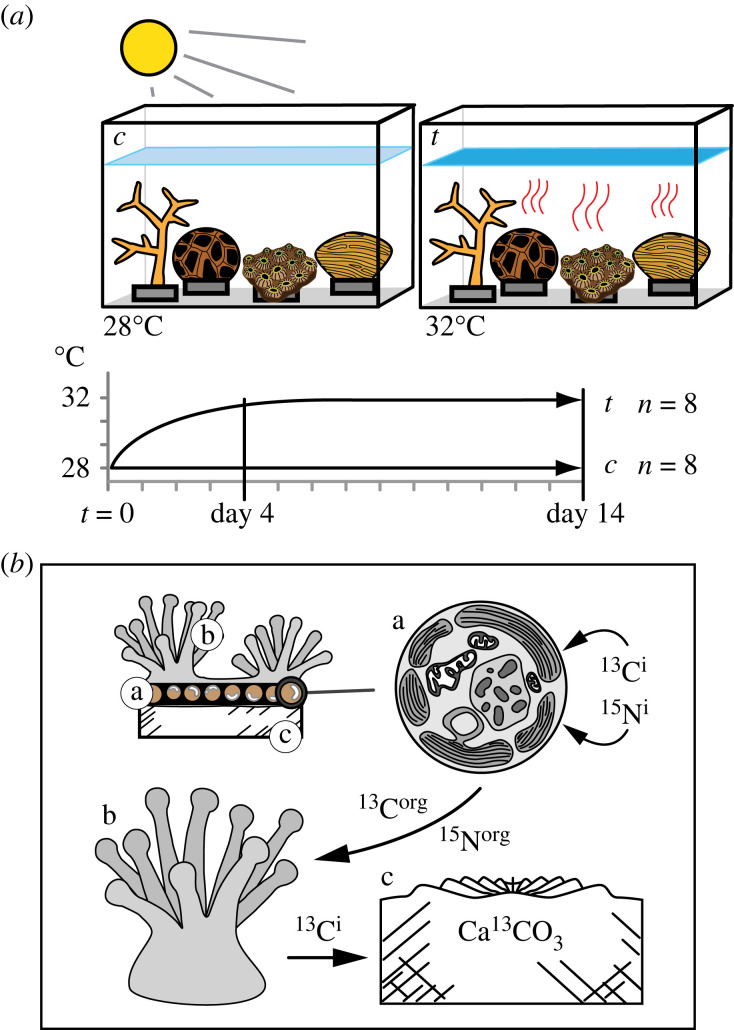
(*a*) Experimental design for thermal stress experiments. Colonies of *Acropora muricata*, *Coelastrea aspera*, *Cyphastrea chalcidicum* and *Pachyseris rugosa* associating with *Durusdinium trenchii* or *Cladocopium* spp. were exposed to thermal stress and compared to clones maintained at 28°C. (*b*) The diagram of the coral–symbiotic dinoflagellate illustrates the three biological compartments targeted in the experiment: (a) symbiotic dinoflagellate, (b) coral tissue and (c) skeleton. The experiment used isotopic enrichment of inorganic carbon (^13^C^i^) and nitrogen (^15^N^i^) by administering $H^{13}{\mathrm{CO}}_{3}^{\text{–}}$ and ${\;}^{15}{\mathrm{NO}}_{3}^{\text{–}}$ following a 14-day experiment at 28°C and 32°C. Inorganic elements were biologically converted into organic compounds (^13^C^org^ and ^15^N^org^) and elemental uptake, assimilation and translocation of isotopic elements were quantified.

For each treatment, two replicate fragments from each coral colony were placed in separate treatment bins. In the heated treatment, the temperature was gradually increased from 27.5°C to 32°C over 4 days, and then maintained at 32°C for an additional 10 days, totalling 14 days of heating. The control treatment was kept at a constant temperature of 27.5°C throughout the 14-day experiment (figure 1). All the experimental coral fragments were kept outdoors, and covered by non-UV filtering clear plastic film (Sun Selector, Ginegar Plastic Products) to protect them from periodic rainfall. Additionally, a 60% shade cloth was used to provide a peak midday light intensity of 800 µmol quanta m^−2^ s*^−^*^1^, measured with a PAR sensor (LiCor LI-192), similar to the maximum light levels of natal colony habitats at collection depth.

To prevent algal fouling, the treatment bins and PVC tiles were cleaned every other day. Additionally, the coral fragments were rotated within their bins every other day to ensure even light exposure and minimize potential tank effects.

At the beginning of the experiment (day 0), one fragment from each coral colony (if available; *n* = 4–8) was removed, and ^13^C and ^15^N isotope measurements (described below) of unlabelled colonies were made and included in figures for enrichment comparison. On day 14 (4 days of temperature ramping and 10 days at 32°C), coral fragments were removed from treatments and processed the same as day 0 (figure 1).

### (c) Photophysiology

A pulse amplitude modulation fluorometer (Diving PAM, Waltz, Germany) was used to measure the maximum quantum yield of photosystem II (PSII, *F*_v_/*F*_m_) 1 h after sunset in three separate locations using a 0.6 s saturation pulse (saturation intensity greater than 1000 µmol quanta m^−2^ s^−1^). Three intracolony *F*_v_/*F*_m_ measurements were averaged together to calculate the mean *F*_v_/*F*_m_ for each fragment. All data were arcsine transformed to meet parametric assumptions and evaluated using a two-way ANOVA comparing the effect of site and temperature.

### (d) Symbiotic dinoflagellate densities

Coral tissue was removed using an airbrush (100 psi) and filtered (0.22 µm) seawater. The resulting slurry, containing coral tissue and symbiotic dinoflagellates, was homogenized for approximately 10 s using a Tissue Tearor (BioSpec Products, Bartlesville, OK, USA). Aliquots (1 ml) were taken from the homogenized slurry and preserved with 1% glutaraldehyde for symbiotic algal enumeration. Algal densities were quantified using an EVOS digital fluorescent microscope from four to six replicate haemocytometer counts (AO Spencer Bright Line Improved Neubauer haemocytometer) and normalized to coral surface area using the aluminium foil method [29] for *C. aspera, C. chalcidicum* and *P. rugosa*, and the hot wax method [30] for the branching coral *A. muricata.* The influence of thermal treatments (32°C) on areal symbiotic dinoflagellate densities were compared to clone fragments at the control temperature (28°C) and the per cent change of symbiotic dinoflagellates were arcsine transformed and evaluated using *t*-tests.

### (e) Inorganic carbon and nitrate uptake

On day 14, control and treatment fragments were placed into glass beakers containing 400 ml of freshly filtered seawater (0.45 µm) that was enriched with 0.633 mM of NaH^13^CO_3_ (99 atom % ^13^C, Cambridge Isotope Lab Inc., Andover, MA, USA), and 1.5 µM of ${\mathrm{Na}}^{15}{\mathrm{NO}}_{3}^{\text{–}}$ (98 atom % ^15^N, Cambridge Isotope Lab Inc., Andover, MA, USA). The background seawater concentrations of dissolved inorganic carbon (DIC) were 1863.3 ± 2.9 µM kg^−1^ [31] and 0.24 ± 0.11 µM of ${\mathrm{NO}}_{3}^{\text{–}}$ as determined using a colorimetric assay as described by the US Environmental Protection Agency (Method 353.2).

Beakers were fitted with false bottoms and continually stirred with magnetic stir bars. All beakers were held constant at the experimental temperatures for 5 h (28°C or 32°C) and illuminated by LED lights (Cree Cool White XP-G R5) set to a light intensity of 500 µmol quanta m^−2^ s^−1^. Preliminary measurements determined this irradiance level was sufficient to maximize photosynthesis (*P*_max_) and the H^13^CO_3_ and ${\;}^{15}{\mathrm{NO}}_{3}^{\text{–}}$ concentrations were sufficient to be used for elemental tracing across the biological compartments. The isotope range for each labelled biological compartment were: symbiotic dinoflagellates AP^13^C 1.73–3.93 or *δ*^13^C 579–2656 and AP^15^N 0.39–1.02 or *δ*^15^N 68–1790, host tissue AP^13^C 1.39–2.44 or *δ*^13^C 48–1241 and AP^15^N 0.37–0.62 or *δ*^15^N 18–696, and coral skeleton AP^13^C 1.13–1.31 or *δ*^13^C 7–183). After isotopic labelling, the fragments were removed, rinsed in filtered seawater and immediately frozen at −60°C. All isotope data were tested for normality using the Shapiro–Wilks and had equal variance. The impact of symbiotic dinoflagellates on the uptake and assimilation of ^13^C and ^15^N across biological compartments was assessed by comparing colonies containing *D. trenchii* with colonies containing *Cladocopium* spp. at a temperature of 28° using *t*-tests. The influence of thermal treatments (32°C) on ^13^C and ^15^N uptake and assimilation across biological compartments, were compared to clone fragments at the control temperature (28°C) using *t*-tests.

### (f) Stable isotope analyses

Coral tissue was removed with an airbrush as previously described, followed by the addition of 0.02% (w/v) sodium dodecyl sulfate (SDS) and homogenization for 10 s with a Tissue-Tearor (Biospec Products, Inc). Symbiotic dinoflagellates and coral tissue were separated by two to three centrifugation washes (550 *g* for 5 min) with 10 s homogenization between each wash [32]. Algal fractions were microscopically verified to ensure the efficiency of the separation technique and to confirm the homogeneity and removal of the bulk animal material [33]. Clean algal cells were pelleted via centrifugation (5000*g* for 5 min) and frozen at −20°C until analysed. Accumulated supernatants (animal portion) were microscopically verified to not contain symbiotic dinoflagellates or skeletal material and were filtered onto pre-combusted (450°C for 5 h) glass 0.7 µm filters (Whatman GF/F) until clogged and frozen at −20°C.

Owing to the relatively high concentration of ^13^C assimilation by the symbiotic dinoflagellates during incubations, coral skeletons were placed in 100% bleach for 24 h to remove any remnant organic material from host-algal tissue, rinsed in freshwater for 24 h, and dried under low heat. Approximately 20 mg of CaCO_3_ was sampled from the corallite and coenosarc regions of the coral skeleton using a Dremel tool with a diamond bit. Skeletal samples were stored at −20°C until analysed. Elemental ^13^C and ^15^N analyses were performed on a Carlo Erba CHN Elemental Analyzer (Model NA1500) coupled to Thermo Finnigan Delta V Isotope Ratio Mass Spectrometer via a Thermo Finnigan Conflo III Interface at the University of Georgia, Center for Applied Isotope Studies. Enriched isotopic data are reported as atom % of the heavy isotope (AP^13^C & AP^15^N) [34].

### (g) Genetic identification of symbiodiniaceae

Symbiont genetic analyses followed the same protocols as detailed in Hoadley *et al*. [17]. Briefly, symbiont DNA was extracted with a modified Promega Wizard genomic DNA extraction protocol [35]. The dominant and co-dominant symbionts inhabiting the experimental coral colonies were identified by two genetic analyses. First, the internal transcribed spacer 2 region (ITS2) was analysed using denaturing gradient gel electrophoresis (DGGE) fingerprinting [36,37]. The dominant bands from each distinctive DGGE fingerprint profiles were excised, re-amplified, and directly sequenced on an Applied Biosciences sequencer (Applied Biosciences, Foster City, CA) at the Pennsylvania State University Genomics Core facility. Therefore, putative species were assigned based on the dominant or co-dominant ITS2 sequence [38]. Second, the nuclear large-subunit ribosomal DNA (LSU) was amplified and sequenced from a subset of samples using methods described by Zardoya *et al*. [39] to verify taxonomic identity. Symbiotic dinoflagellate taxonomic designation were assigned as described in Butler *et al*. [40].

## 3. Results

### (a) Host–symbiont associations and bleaching response

We used the common physiological measurements of symbiotic dinoflagellate densities and the maximum photochemical efficiency of PSII (*F*_v_/*F*_m_) to detect a physiological effect of heating and compare the influence of thermal stress on corals associating with either *D. trenchii* or *Cladocopium* spp.

**Figure 2.**
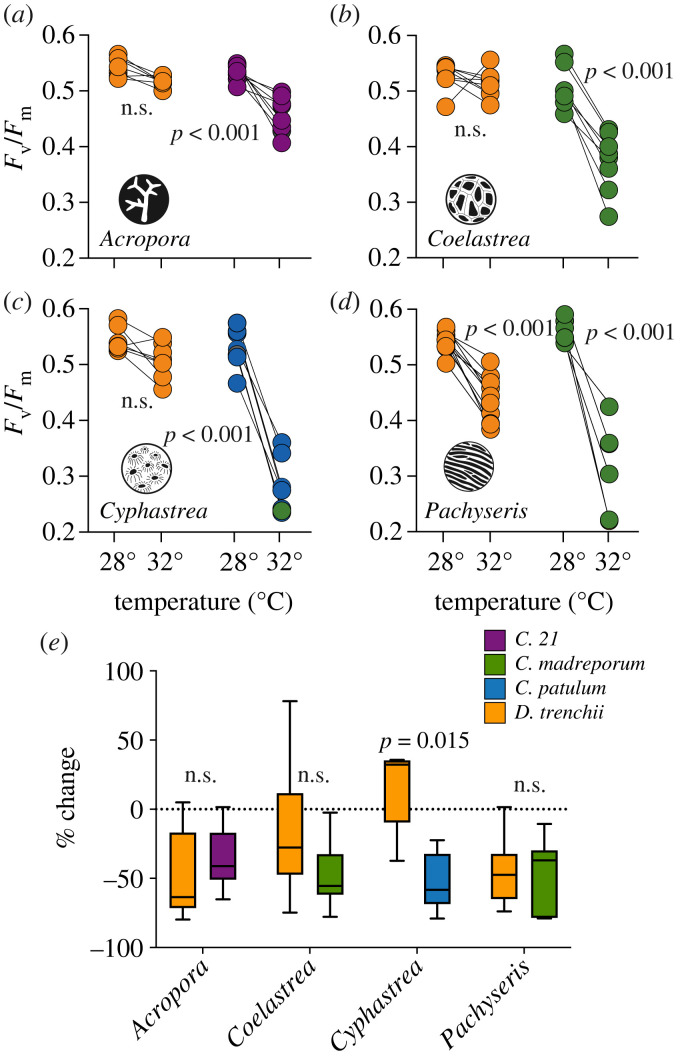
Physiological influence of thermal stress after a 14-day experiment (*n* = 6–8). The maximum photochemical efficiency of PSII (*F*_v_/*F*_m_) was measured on day 14 for colonies with *Durusdinium trenchii* or *Cladocopium* spp. as a function of temperature. Each connected point is from clone fragments with points at 28°C and 32°C after 14 days. (*a*) *Acropora muricata.* (*b*) *Coelastrea aspera.* (*c*) *Cyphastrea chalcidicum.* (*d*) *Pachyseris rugosa.* (*e*) Per cent change of areal symbiotic dinoflagellate densities of conspecific coral species with *D. trenchii* or *Cladocopium* spp. symbionts after a 14-day of heating at 32°C. Different colours correspond with the species or ITS2 type of symbiotic dinoflagellate associated with each colony. Significant *p*-values are listed adjacent to the data (*t*-test, d.f. = 1).

Overall, *F*_v_/*F*_m_ was similar between conspecific corals with *D. trenchii* or *Cladocopium* spp. at 28°C, however, photosynthetic capacity was suppressed with temperature. A two-way ANOVA was performed to analyse the effect of symbiont association and temperature on the maximum photochemical efficiency of PSII (*F*_v_/*F*_m_). A two-way ANOVA revealed that there was a statistically significant interaction between symbiont association and temperature for each species (table 1). Using post-hoc analyses we found, with the exception of *P. rugosa*, that all coral species with *D. trenchii* maintained *F*_v_/*F*_m_ equivalent to the controls during the temperature treatment, while *F*_v_/*F*_m_ significantly declined during the temperature treatment in all colonies that harboured *Cladocopium* spp., regardless of coral species (figure 2*a–d*; Tukey test: *p* < 0.05).

**Table 1.** Summary of two-way ANOVA statistics to test the effects of symbiotic dinoflagellate association and temperature treatment for maximum photochemical efficiency of PSII (*F*_v_/*F*_m_) on *Acropora muricata*, *Coelastrea aspera*, *Cyphastrea chalcidicum* and *Pachyseris rugosa*. Statistically significant *p*-values (less than 0.05) are indicated by italics.

source of variation	d.f.	SS	*F*	*p*-value
*Acropora*				
symbiont (S)	1	0.009	21.788	*<0.001*
temperature (T)	1	0.020	47.825	*<0.001*
S × T	1	0.005	11.622	*0.002*
residual	27	0.011		
total	30	0.047		
*Coelastrea*				
symbiont (S)	1	0.053	37.744	*<0.001*
temperature (T)	1	0.045	31.851	*<0.001*
S × T	1	0.029	20.755	*<0.001*
residual	28	0.039		
total	31	0.166		
*Cyphastrea*				
symbiont (S)	1	0.122	89.729	*<0.001*
temperature (T)	1	0.167	122.960	*<0.001*
S × T	1	0.093	68.391	*<0.001*
residual	27	0.037		
total	30	0.424		
*Pachyseris*				
symbiont (S)	1	0.006	1.811	0.189
temperature (T)	1	0.210	65.158	*<0.001*
S × T	1	0.020	6.030	*0.021*
residual	28	0.090		
total	31	0.326		

After heating at 32°C, symbiotic dinoflagellate number was lower by approximately 50% in most colonies than in ramets kept at 28°C (figure 2*e*). Regardless of symbiotic dinoflagellate association, conspecific colonies lost similar amounts of their symbiotic algae (figure 2*e*; *t*-test: *p* > 0.05) One exception to this pattern was for *C. chalcidicum* that associated with *D. trenchii* where symbiont densities remained the same as control values after heating (figure 2*e*).

### (b) Inorganic carbon assimilation by symbiotic dinoflagellates

At 28°C, ^13^C assimilation by symbiotic dinoflagellates remained largely similar between coral colonies hosting *D. trenchii* and *Cladocopium* spp. (electronic supplementary material, figure S1; *t*-test *p* > 0.05). However, there was a distinction in ^13^C assimilation between *D. trenchii* and *Cladocopium* spp. in *C. chalcidicum*. Colonies containing *D. trenchii* exhibited notably higher ^13^C uptake compared with those with *Cladocopium* spp. (electronic supplementary material, figure S1; *t*-test *p* < 0.05).

**Figure 3.**
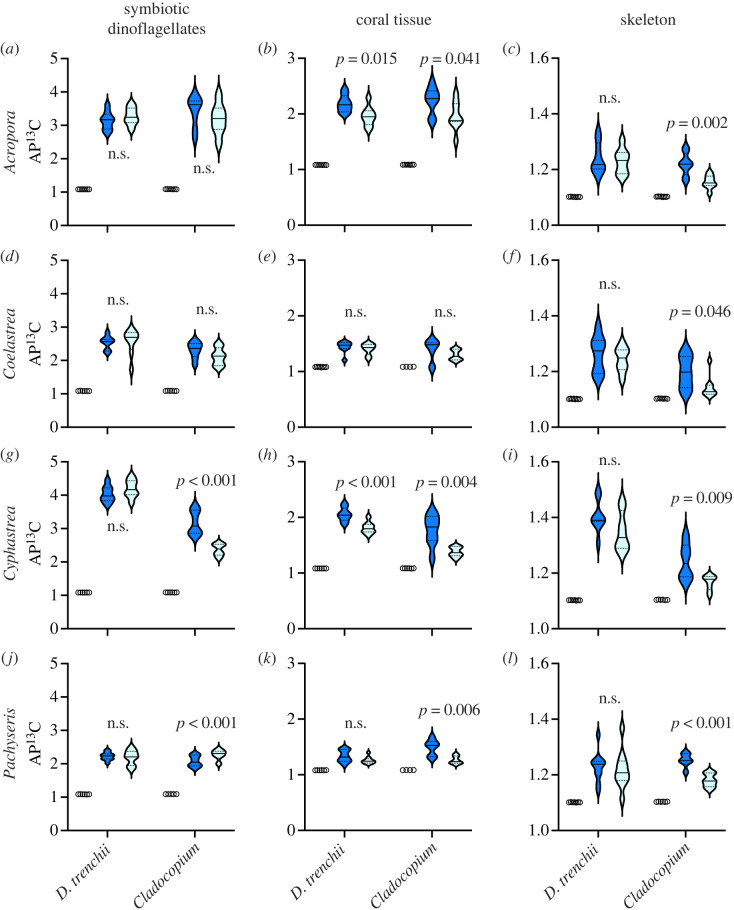
Atom % ^13^C (AP^13^C) enrichment after a 5 h pulse of $H^{13}{\mathrm{CO}}_{3}^{-}$ following 14 days at ambient (28°C) (dark blue) and elevated (32°C) (light blue) temperatures. AP^13^C was measured from symbiotic dinoflagellates, host tissue and coral skeleton. The natural abundance of AP^13^C from each species and host–symbiont association are included as open circles (*n* = 4–8). Conspecific colonies associated with *Durusdinium trenchii* or *Cladocopium* spp. symbionts as indicated on *x*-axis. (*a–c*) *Acropora muricata.* (*d–f*) *Coelastrea aspera.* (*g–i*) *Cyphastrea chalcidicum.* (*j–l*) *Pachyseris rugosa.* Violin plots are kernel densities and depict the median and first and third quartiles (*n* = 6–8). Significant *p*-values are listed adjacent to the data and compare the effects of temperature on clone fragments (*t*-test, d.f. = 1).

After 14 days of heating, inorganic carbon uptake between control and heated samples was similar in all colonies with *D. trenchii*, as well as in *Cladocopium* spp. within *A. muricata* (figure 3*a,d,g,j*). By contrast, inorganic carbon assimilation was significantly lower at 32°C than at 28°C in *C. chalcidicum* with *C. patulum* and *C. madreporum* (figure 3*g*; *t*-test: *p* < 0.001).

### (c) Coral photosynthetic ^13^C tissue incorporation and skeletal deposition

Host tissue and skeleton ^13^C incorporation remained largely similar between coral colonies hosting *D. trenchii* and *Cladocopium* spp. at 28°C (electronic supplementary material, figure S1; *t*-test *p* > 0.05). However, ^13^C was different between colonies harbouring *D. trenchii* and *Cladocopium* spp in *C. chalcidicum*, where colonies with *D. trenchii* had significantly greater ^13^C assimilation and incorporation in the host tissue and skeleton (electronic supplementary material, figure S1; *t*-test: *p* < 0.05).

While coral tissue ^13^C was significantly reduced at 32°C in *A. muricata* and *C. chalcidicum* with *D. trenchii*, this loss in C-incorporation with heating was even lower in these corals with *Cladocopium* spp. (figure 3*b,h*; *t*-test: *p* < 0.05). ^13^C carbon transfer to host tissue diminished significantly relative to controls in all coral species harbouring *Cladocopium* spp. except for *C. aspera* where ^13^C enrichment remained similar between the two temperatures (figure 3*b,e,h,k*; *t*-test *p* < 0.05).

Temperature had no effect on inorganic carbon (^13^C) skeleton deposition in all colonies with *D. trenchii* (figure 3*c,f,i,l*). By contrast, all colonies with *Cladocopium* spp. held at 32°C had significantly lower skeletal ^13^C values than 28°C control treatments (figure 3*c,f,i,l*; *t*-test: *p* < 0.05).

### (d) Nitrate uptake and ^15^N assimilation by symbiotic dinoflagellates

**Figure 4.**
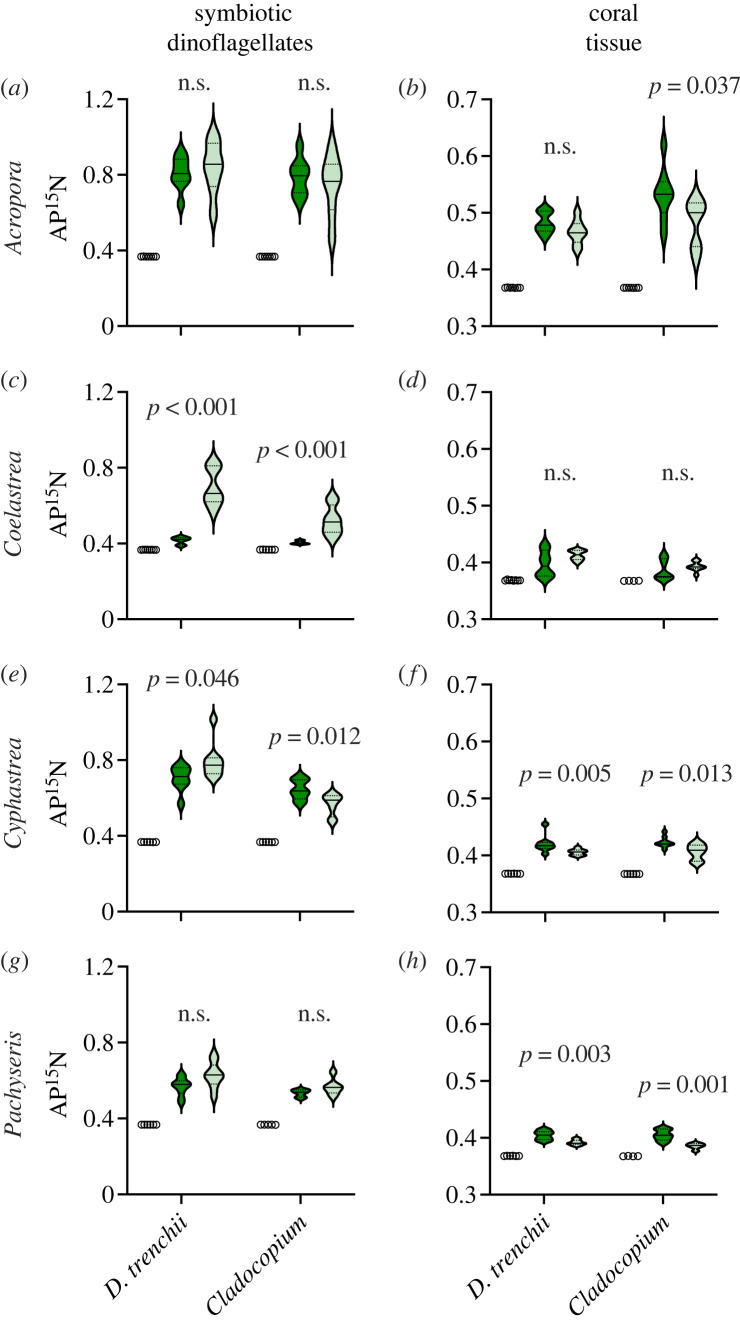
Atom % ^15^N (AP^15^N) enrichment after 5 h pulse of ${\;}^{15}{\mathrm{NO}}_{3}^{\text{–}}$ following 14-days at ambient (28°C) (dark green) and elevated (32°C) (light green) temperatures. AP^15^N was measured from symbiotic dinoflagellates and host tissue. The natural abundance of AP^15^N from each species and host–symbiont association are included as open circles (*n* = 4–8). Conspecific colonies associated with *Durusdinium trenchii* or *Cladocopium* spp. symbionts as indicated on *x*-axis. (*a,b*) *Acropora muricata.* (*c,d*) *Coelastrea aspera.* (*e,f*) *Cyphastrea chalcidicum.* (*g,h*) *Pachyseris rugosa.* Violin plots are kernel densities and depict the median and first and third quartiles (*n* = 6–8). Significant *p*-values are listed above data and compare the effects of temperature on clone fragments (*t*-test, d.f. = 1).

At 28°C, ^15^N uptake by symbiotic dinoflagellates remained similar between coral colonies hosting *D. trenchii* and *Cladocopium* spp. (electronic supplementary material, figure S1; *t*-test *p* > 0.05). However, after the 32°C temperature treatment there were mixed responses found in ^15^N incorporation across coral taxa. No effects of temperature on NO_3_^–^ uptake were detected in *A. muricata* or *P. rugosa* regardless of *Cladocopium* spp. or *D. trenchii* symbioses (figure 4*a,g*). During heating, all *C. aspera* colonies, regardless of symbiotic dinoflagellate association, were found to significantly increase the uptake of NO_3_^–^ than clones maintained at the control temperature of 28°C (figure 4*c*; *t*-test: *p* < 0.001). *Cyphastrea chalcidicum* colonies with *D. trenchii* also had significantly greater symbiotic algal NO_3_^–^ uptake during thermal stress (figure 4*e*; *t*-test, *p* < 0.05); however, NO_3_^–^ uptake significantly declined in *C. chalcidicum* with *Cladocopium* spp. at 32°C (figure 4*e*; *t*-test, *p* < 0.05).

### (e) Nitrogen incorporation into coral tissue

There were no differences detected in ^15^N assimilation in coral tissue between coral colonies with *D. trenchii* or *Cladocopium* spp. at 28°C. Fourteen-day exposure to 32°C decreased ^15^N assimilation to host tissue in *C. chalcidicum* and *P. rugosa* with *D. trenchii* (figure 4*f,h*; *t*-test: *p* < 0.05) and no differences were detected in *A. muricata* or *C. aspera* with *D. trenchii* (figure 4*b,d*). Host tissue ^15^N was significantly reduced in all heated colonies with *Cladocopium* spp. (figure 4*b,f,h*; *t*-test: *p* < 0.05) except for *C. aspera*, which remained similar to the control temperature (figure 4*d*).

### (f) Symbiotic dinoflagellate identification

All corals from the near-shore habitat were found to contain only *D. trenchii*, as confirmed through ITS2 screening and LSU rDNA sequencing. In offshore corals, *C. aspera* and *P. rugosa* contained *C. madreporum*, however, in two colonies of offshore *P. rugosa*, *D. trenchii* were observed as the dominant symbiont. Offshore colonies of *A. muricata* harboured *Cladocopium C21*, and most colonies of *C. chalcidicum* contained *C. patulum* as the dominant symbiont with a single colony that contained *C. madreporum*. The dominant symbiont remained consistent in all corals and treatments throughout the experiment.

## 4. Discussion

Carbon and nitrogen assimilation and transport in host colonies with *D. trenchii* and colonies with *Cladocopium* spp. were similar at ambient seawater temperatures (figure 3*a–h*, S1). This equivalent performance was unexpected given how past comparative experiments established that thermally tolerant mutualisms involving *Durusdinium* came at the expense of growth and calcification [23–27,41] (but see Grottoli *et al*. [42] and Turnham *et al*. [43]. Consistent with this observation, comparative studies conducted on corals from the same reef habitats, which examined colony biomass, energy reserves, symbiont cell densities and photobiology, have shown that conspecifics from offshore and near-shore populations are physiologically similar despite having different dinoflagellate genera and species [44,45]. A large disparity in functionality between these mutualisms only emerged once thermal stress was applied (see discussion below). Ultimately, thermally tolerant mutualisms may also sustain or promote coral growth under certain environmental contexts, especially in warm regions where these mutualisms have long coexisted.

While the efficiency of PSII reaction centers (*F*_v_/*F*_m_) declined in all colonies after 14 days of thermal stress, photochemical loss was considerably greater in all colonies harbouring *Cladocopium* spp. (figure 2*a–d*). This observation is consistent with a large body of field and experimental work showing that colonies with *Durusdinium* tend to tolerate physiological stress better than other partnerships [5,8,17,20,46]. However, explanations for reduced *F*_v_/*F*_m_ can differ among symbionts. For some, it is a clear proxy for photodamage [47], but for others, it may correspond with the temporary downregulation of functional Photosystem II reaction centres [48,49]. That is why measurement of cell densities relative to control offered an additional indicator of a mutualism's physiological condition.

At 32°C, most mutualisms were under stress, as indicated by the proportional loss of symbionts (figure 2*e*). Although *D. trenchii* retained a higher *F*_v_/*F*_m_ than *Cladocopium* spp. in conspecific host colonies, there was no relationship between symbiotic dinoflagellate identity and algal loss with heating in three of the tested coral taxa (figure 2). However, one notable exception was for *Cyphastrea*, where *D. trenchii* densities remained similar at 32°C and 28°C (figure 2). These findings corroborate previous observations that *Cyphastrea* are better able to tolerate high-temperatures than other coral taxa [50–52]. Such thermal tolerance may be due to a mutually beneficial relationship with *D. trenchii*.

The uptake of inorganic carbon and the translocation of organic carbon are critical processes in the physiology of reef-building corals. They play an imperative role in enhancing coral reef productivity and ensuring their long-term persistence [3,53,54]. In this experiment, the ability of *D. trenchii* in all hosts, *Cladocopium* C21 in *Acropora* and *C. madreporum* in *Coelastrea* to assimilate DIC (i.e. CO_2_) after 14 days of thermal stress were similar to controls. Notable exceptions were the precipitous decline, in AP^13^C in *Cladocopium patulum* in *Cyphastrea* at high-temperatures, whereas carbon fixation increased in colonies of *Pachyseris* with *C. madreporum* (figure 3). Thus, with one exception, inorganic carbon assimilation was maintained by most symbiont species.

The application of heat stress revealed significant differences in nutrient translocation, offering insights into the stability of host–symbiont partnerships under physiological challenges. While most symbionts maintained stable carbon assimilation, host colonies harbouring *Cladocopium* spp. (except for Coelastrea) experienced marked reductions in carbon translocation. This difference between symbiont ^13^C and host tissue ^13^C relative to controls indicates a disruption in the transfer of photosynthetic products (e.g. carbohydrates) from symbiont to host. The signal of diminished carbon flow was further amplified in the skeleton, as colonies associated with *Cladocopium* spp. exhibited a significant decline in mean skeletal carbon assimilation (figure 3). In marked contrast; however, skeletal carbon incorporation was unaffected by thermal stress in colonies associated with *D. trenchii*, indicating that the carbon translocation from symbiont to host tissue and subsequent metabolism into the skeleton remained stable during thermal stress. This difference between *Durusdinium* colonies and *Cladocopium* colonies has important implications for understanding how metabolic processes influence differences in the thermal tolerance of these mutualisms [16,17].

Prolonged temperature stress can also disrupt the transfer of the symbiont-derived organic nitrogen to the host, further destabilizing the mutualism [55,56]. Indeed, less ^15^N was assimilated into coral tissue during thermal stress regardless of symbiont identity in all but one coral species (*Coelastrea*) that increased ^15^N assimilation with heating when harboring *D. trenchii* (figure 4). The rapid acclimation response to high-temperature stress, and associated cellular damage, raises the demand for nitrogen needed for increased production, modification, and/or repair of proteins and lipids [47,56,57]. Nitrogen assimilation in cells of *D. trenchii* was either unaffected or increased depending on the host species in which it occurred (figure 4). Similarly nitrogen assimilation by *Cladocopium* spp. varied depending on host identity but diminished significantly in *C. patulum* from *Cyphastrea* (figure 4). While thermal stress had a minimal or increased effect on nitrogen assimilation by symbiotic dinoflagellates, with one notable exception (i.e. *Cyphastrea*; figure 4*e*), as with carbon, nitrogen transfer to the host tissue was more adversely affected (figure 4*b,f,h*). These results demonstrate that *D. trenchii* often assimilates nitrogen at higher temperatures relative to *Cladocopium* spp. Assuming the model of nitrogen movement by Pernice *et al*. [58], after rapid acquisition of inorganic N by the symbiont, the subsequent transfer of organic nitrogen from *D. trenchii* to the host remains stable or is reduced, depending on the host partner (figure 4*b,d,f,h*). The physiological advantages of maintaining nitrogen assimilation and transfer may explain, in part, the resiliency of these mutualisms during episodic heatwaves [3,55]. Excess nitrogen pollution from agricultural and sewer runoff, however, can alter important cellular processes, disrupt host–symbiont nutrient exchange and make corals more thermally susceptible [59,60], but these external factors do not apply here. Ultimately, gauging the internal cycling of nutrients between host and symbiont is vital in assessing a colony's response and resiliency to environmental change [3,61,62].

The correspondence between physiological stress and diminished nutrient transfer between symbiont and host further supports ideas that disruptions to carbon and nitrogen cycling play an important role in the breakdown of thermally sensitive partner combinations [3,63,64]. The diminished transfer of carbon and nitrogen from symbiont to the host, and resulting engergetic deficit, probably has a destabilizing effect on the mutualism (fig. 4 in [65]). Retaining photosynthetic function and the capacity to transfer carbon and nitrogen is clearly important for the persistence of these mutualisms. In this regard, the continued physiological function of *D. trenchii* under thermal duress probably helps to stabilize its mutualisms with these Pacific Ocean corals [17].

How do these findings differ from previous conclusions regarding associating with thermally tolerant symbionts like *Durusdinium*, that resulted in reduced productivity [24–27]? Notably, physiological trade-offs in hosts dominated by *Durusdinium* were primarily studied in Acropora colonies from the Great Barrier reef where these mutualisms are not as common as in the near-shore reef communities of Palau [66,67] or where the symbiont was recently introduced [26]. Though widespread throughout the west Indo-Pacific, the prevalence and diversity of symbionts in the genus *Durusdinium* are most abundant in the equatorial regions around the Indo-west Pacific (e.g. [5]). This diversity appears to be a product of a recent adaptive radiation during the Pleistocene [19,68]. Thus, prevalent mutualisms involving *Durusdinium* from equatorial lagoonal environments likely evolved to thrive in reef environments that are inhospitable to many other host–symbiont combinations. Indeed, for corals where the host and symbiont have co-evolved, metabolic trade-offs affecting colony productivity is not apparent [43].

## 5. Conclusion

While these observations are limited to the short-term uptake and assimilation of C and N during acute thermal stress experiments, it may be concluded that symbioses co-evolved to live in warm water habitats exhibit high functionality over a range of thermal conditions. The finding of high nutrient assimilation and translocation under normal and thermally stressful conditions helps to explain why corals with *D. trenchii* thrive in warmer water environments. The lack of apparent physiological trade-offs requires further investigation to determine the seasonal and long-term physiological performance of these mutualisms and the tracking of fitness proxies such as colony growth and gamete production. Nevertheless, the data presented here support the contention that corals associated with *Durusdinium* do not necessarily experience significant physiological trade-offs during non-stressful periods and retain greater physiological function at increased temperatures. As mutualisms adapted to thriving in less hospitable reef environments, the proliferation and spread of *Durusdinium* spp. may play an essential role in reef coral persistence and growth as oceans continue to warm [69].

## Data Availability

The data provided in electronic supplementary material [70] or at www.bco-dmo.org and can be accessed by doi:10.26008/1912/bco-dmo.907003.1.
